# Weakened PAM/PEI Polymer Gel for Oilfield Water Control: Remedy with Silica Nanoparticles

**DOI:** 10.3390/gels8050265

**Published:** 2022-04-24

**Authors:** Zulhelmi Amir, Ismail Mohd Saaid, Mohd Usman Mohd Junaidi, Wan Zairani Wan Bakar

**Affiliations:** 1 Department of Chemical Engineering, Faculty of Engineering, Universiti Malaya, Kuala Lumpur 50603, Malaysia; usmanj@um.edu.my; 2 Center for Energy Science, Universiti Malaya, Kuala Lumpur 50603, Malaysia; 3 Engineering Department, Universiti Teknologi PETRONAS, Bandar Seri Iskandar 32610, Perak, Malaysia; ismailsaaid@utp.edu.my; 4 Oil & Gas Department, School of Chemical Engineering, College of Engineering, Universiti Teknologi MARA, Shah Alam 40450, Selangor, Malaysia; zairani@uitm.edu.my

**Keywords:** polymer gel, water control, silica NP, gel strength, coreflooding

## Abstract

Polymer gel treatment is one of the most popular conformance control methods used in the petroleum industry. The advantage of the polymer gel system used in harsh reservoir conditions is an integrated process that must take into account all elements of gelation kinetics. In high-temperature applications, NH_4_Cl has been selected as a retarder to extend the gelation time of a PAM/PEI gel system. However, the gel network loses gel strength when salt and retarder increase, resulting in a weak gel structure, and becomes susceptible. The combination of these two variables leads to the development of a weak gel network, making it fragile and susceptible. To strengthen the weakened PAM/PEI polymer gel, the addition of silica nanoparticles (silica NP) is considered an effective remedy. This article presents the performance of PAM/PEI polymer gel strengthened with silica NP, especially the performance in terms of viscosity, gelation time, and gel strength, as well as performance in porous media. For example, the results exhibited a high storage modulus, G′, which is almost 800 Pa, compared to the loss modulus, G″, throughout the frequency and strain range, indicating solid-like behavior, at significantly high amounts of silica NP. This finding provides a better understanding and knowledge on the influence of solid particles in enhancing the performance of PAM/PEI polymer gel that has been weakened by salinity and retarder.

## 1. Introduction

A perfect oilfield conformance facilitates a consistent sweep of the hydrocarbon across the whole reservoir towards the production well. However, in many cases, excessive water production during water flooding occurs due to conformance problems [1]. The most common root cause of conformance problems is the formation of heterogeneous or stratified reservoirs with a high permeability streak [2]. When injecting water into a reservoir, injected water flows through its easiest pathway, in the relatively higher permeability region. As a result, the oil is bypassed by water in the high-permeability zone. In fact, it leaves behind large portions of the unswept oil in the region with relatively lower permeability, thus producing more water rather than oil [3]. In this way, it can be understood that high produced water is related to inefficient oil sweep [4].

Water is the most plentiful fluid in the oilfield. Water could bring significant advantages, but abundant produced water is not required in the oil and gas industry. Produced water is produced in the wellbore without additional oil recovery or sufficient amounts of oil to cover the cost of water handling. Excessive water production is unfavorable and poses a huge challenge as it hinders high oil production, with an increase in operational costs for water treatment, thus generating low profitability [5]. Worldwide daily water production is estimated at 310 million barrels from offshore and onshore oilfields, with 78 million barrels of oil per day [6]. It is the same as bringing four barrels of water to the surface with every barrel of oil. According to these numbers, oil companies may be able to shift their business to the water industry.

Produced water has been in contact with the hydrocarbon-bearing formation for centuries. Thus, it has some of the chemical characteristics of the formation and the hydrocarbon itself. The treatment of produced water is of paramount importance to the oil and gas industry due to legislation, environmental factors, and sustainability, as well as reducing contamination of fresh water sources. It also has been reported that the costs of disposing and treatment of produced water are high. The cost can be as high as USD 5 per barrel of water, depending on the technologies that are used. Taking USD 0.5 per barrel of water as the nominal disposal cost, the worldwide oil and gas sector must endure an enormous amount of nearly USD 55 billion in annual costs for managing 310 million barrels of water per day [6,7]. When the expense of handling produced water surpasses the value of the hydrocarbon produced by the well, well shutdown could be inevitable. Due to this, reducing water production has gained much attention from the oil and gas industry.

**Figure 1:**
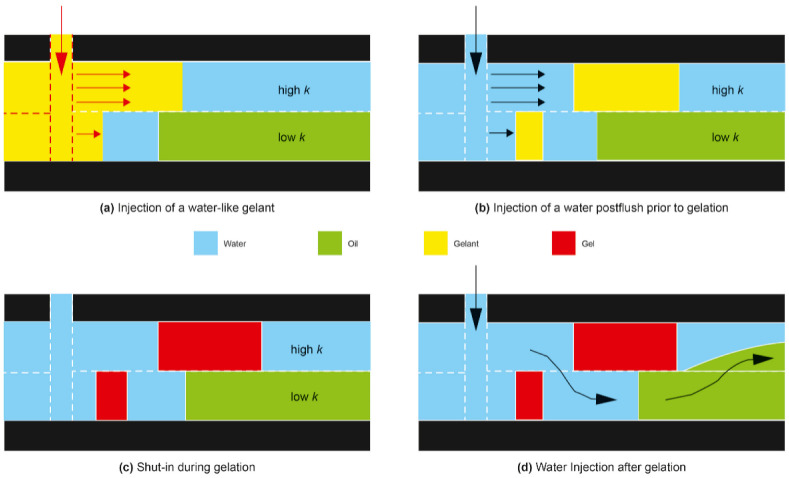
The concept of placement and formation of gel for conformance control with crossflow effect. Figure from [12].

One of the characteristics of hydrocarbon reservoirs is having layered permeability formation ranging from the high-permeability (high k) to low-permeability (low k) region. Water control can be effectively conducted by reducing the permeability of the high-permeability region of the reservoir [8]. Because of the permeability modification, the new subsequent water injection will flow into the “higher permeability” zone, where the residual oil is located [5]. The idea of gel placement into reservoirs has been established by Seright et al. in 2012, as shown in Figure 1 [9]. A gel-based approach has been shown to be applicable for most reservoirs, which require conformance control through permeability reduction [10]. In-situ crosslinked polymer gels are developed to travel through fractures and layers with high permeability and water saturation. Following well shut-in to allow gelation, a polymer gel helps in the formation of a barrier to prevent water penetration and, consequently, reduces the flow of undesired water [11]. The subsequent water injection will flow with crossflow effect across the previously unswept low-permeability zones, resulting in improved oil production.

**Figure 2:**
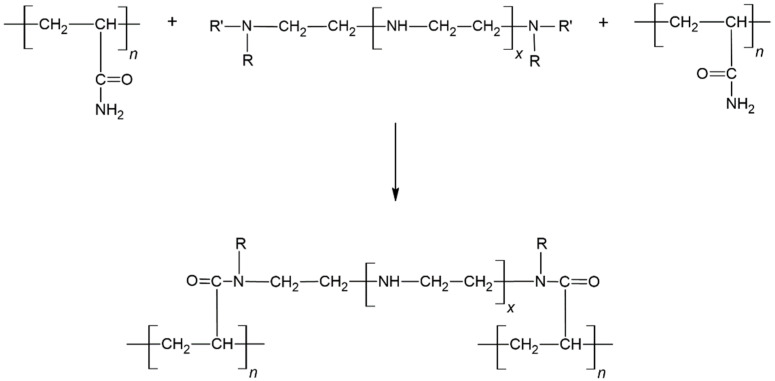
Crosslinking reaction through a transamidation reaction [13].

One of the most prevalent organically crosslinked polymer gels used for water control is polyacrylamide (PAM) and polyethylenimine (PEI)-based crosslinked polymer gel. PAM and PEI crosslinking can be defined as a transamidation process, in which the imine nitrogen in PEI replaces the amide group at the carbonyl carbon of PAM [13]. This mechanism is shown in Figure 2. Then, the use of saltwater or partly desalinated seawater may be the sole sustainable alternative for preparing polymer solutions in some isolated places, particularly offshore in South East Asian countries, the Arabian and Mexican Gulfs, and the North Sea. Salinity has the ability to either accelerate or delay the gelation process. Furthermore, in order to avoid early gelation and ensure deep penetration of the injected gelant into the required zone, researchers have recommended the optimal concentration of retarders to improve gelation time in the last several years [8]. The retarder has an effect on the crosslinking reaction, which prolongs the time it takes to form the gel [14]. In this case, for high-temperature conformance control, NH_4_Cl was selected as a retarder to extend the gelation time of PAM/PEI polymer gel. However, the combination of salinity and retarder affects the hydrodynamic volume of the polymer, thus limiting the amount of accessible sites on the polymer and contributing to the inadequate gel strength [15]. It can be denoted the formation of a weak gel network, making it fragile and susceptible. A weak gel leads to less effectiveness in blocking and diverting waterflooding to the zone where trapped oil is located. Hence, excessive water production problems still occur and challenge the profitability of the oilfield. Because of these disadvantages, solid particles are introduced to improve the gel strength.

The addition of silica nanoparticles (silica NP) is also shown as a simple and economical solution as an additive for polymer gel in conformance control and wellbore strengthening applications [16]. There is also a study on the influence of silica NP on the gelation behavior of HMTA-HQ crosslinked polymer gel by Liu et al. [17]. The result shows that silica NP considerably improves the gel’s elasticity and viscosity. Because of the hydrogen bonding between silica NP and polymer, its presence enables the gel’s rheological characteristics to become more solid-like. During the crosslinking reaction, silica NP aggregated and formed arrangements in a certain manner. Nanoparticle aggregations generally happen on polymer chain bunches and gel structure meshes. This contributes to a significant increase in gel strength. The introduction of silica NP also notably improved the gel’s thermal stability. This is owing to the water-lockup function related to the great amount of hydroxyl groups on the silica NP surface [17]. Furthermore, silica NP has negative charges in the gelling solution. With water molecules, the hydroxyl groups form hydrogen bonds and electrostatic attractions. This causes the water molecules to become entangled. A higher bound water ratio suggests a stronger gel since it has a higher water holding capacity and thermal stability. Studies using silica NP as an additive also demonstrated promising results in enhancing the polymer gel’s salt tolerance. The works by Metin et al. emphasized three strategies involving the usage of silica NP to reinforce polymer gels [18]. First, the silica NP is in the form of colloidal silica, which is a liquid suspension of fine amorphous, nonporous, and spherical silica particles, rather than a sodium silicate powder; second, the gelation is controlled by salinity differences rather than being triggered by pH changes; and finally, gelation occurs at very low silica NP concentrations.

The performance of weakened PAM/PEI polymer gel that can be strengthened with silica NP is noteworthy to investigate, thus becoming the main objective of this paper. This is a fine-tuning of the gel strength, which has been identified as the disadvantage of PAM/PEI polymer gel containing NH_4_Cl in high salinity, as has been found from previous studies.

## 2. Materials and Methods

### 2.1. Materials

In this study, the components to form gel are non-ionic polyacrylamide (PAM) as the polymer and polyethylenimine (PEI) as the crosslinker. The materials were obtained from Sigma Aldrich and BASF, respectively. PAM is in granular form with pH 6 and the molecular weight of PAM is on average 5,000,000–6,000,000 mol. wt. Meanwhile, the pH of PEI is approximately 11.7, in liquid form, with a molecular weight of 35,000 mol. wt. and active content of 99%. PAM and PEI were used as received, without any further treatment for gel preparation. Other materials were sodium chloride (NaCl) to represent salinity, ammonium chloride (NH_4_Cl) to act as a retarder, and silica nanoparticles (silica NP) as a solid filler to strengthen the polymer gel. All of the salts were analytical reagents of American Chemical Society grade that were purchased from Sigma-Aldrich (M) Sdn Bhd, Petaling Jaya, Malaysia. According to the supplier, the diameter of the silica NP was, on average, 5 nm. To ensure that silica NP had suitable strength, the particle surface was spherical and solid without pores. Deionized water was from our laboratory.

### 2.2. Preparation of Polymer Solution

To prepare polymer gelant samples, 1.5 wt% of PAM and 0.3 v% of PEI were mixed at room temperature. The concentrations of PAM and PEI were from a formulation by Amir et al. [19]. As the optimal gelation process is in neutral conditions, hydrochloric acid (HCl) was added to neutralize the pH of the mixture. The solution was mixed at room temperature with an appropriate mixing time and speed to avoid shear degradation in the polymer gelant samples. By retaining the PAM and PEI concentrations, salinity and retarder were fixed at 30,000 ppm and 5 wt%, respectively, based on the results from a previous study by Amir et al. [15]. Moreover, the solid content, which was silica NP addition, varied in the range of 0.05–1.0 wt% for the subsequent experiments. Silica NP was added first, followed by adding polymer and other materials. After stirring for 30 min, an orbital shaker followed by an ultrasonic bath were used to ensure that the nanoparticles were distributed evenly and to prevent agglomeration.

### 2.3. Measurements

#### 2.3.1. Gelation Time

Several approaches for assessing gelation kinetics have been suggested in the literature. Some of the usually used methods include the bottle test, sealed tube, and the dynamic or static shear methods. The static shear method was chosen in this study to investigate the effect of various raw material concentrations on the gelation time and viscosity changes in the polymer gel. The method was selected as it provides a quantifiable evaluation of gelation time, gelation rate, and gel viscosity. Additionally, compared to the other methods, this method is practically easier to obtain the result, more accurate, and cost-efficient. This method has also been utilized in some papers to determine gelation time [20,21,22]. To determine the viscosity and gelation time, graphs of viscosity changes versus measurement time were used. The inflection point of the curve was used to identify the gelation point.

An Anton Paar RheolabQC rotating rheometer was used to assess viscosity changes and gelation time in this study. This rheometer consists of a sample cup, cylindrical sample container, measurement spindle, temperature control device, and insulating element. The gelling solutions were made and put in the sample cup for each measurement. The sample was then placed in a sample container that had been heated to the desired temperature. The measurements were made at 95 °C and at atmospheric pressure. The temperature was controlled using the Peltier temperature device, which was coupled to the rheometer. The measuring spindle was then immersed in the sample, and the shear rate of 10 s^−1^ was maintained during the test, which is a typical shear rate in rock formation. The measuring spindle in the sample cup was constantly stirring the sample during the measurement. This procedure was used to ensure that the temperature was uniform throughout the sample. Rheoplus software was used to automatically calculate viscosity data, and the acquired viscosity data were recorded in relation to experimental time.

#### 2.3.2. Viscosity and Gel Strength

Viscometry and oscillation experiments were done to determine the gel strength and viscosity of PAM/PEI gel with the influence of salinity, retarder, and solid filler.

a. Viscometry Test

The viscometry test was used to investigate the viscosity and flow behavior of the tested fluid as a function of shear rate. An Anton Paar RheolabQC rotating rheometer with the connection to the measurement devices was used in this investigation. It is a rotating rheometer that applies controlled shear deformation to a fluid sample. The device measured the viscosity of the applied fluid by rotating the spindle at a specified rotational velocity to provide the requisite shear rates. The viscosity was calculated using Newton’s Law based on the evaluated force of resistance imposed on the spindle. In this investigation, the flow and viscosity of polymer solutions were measured at shear rates ranging from 1.0 to 1000 s^−1^. Because the relationship between viscosity and shear rate is frequently associated with flow, the flow behavior of the fluid was illustrated by presenting it as viscosity against shear rate.

b. Oscillation Test

The oscillation test offers important data on the viscoelasticity and strength of polymer gels. For oscillation or gel strength testing, a TA Instrument oscillation rheometer equipped with a parallel plate geometry measurement system was employed. The gelant samples were placed in the oven at 120 °C for at least three days before this test, or whenever the sample was seen to have acquired the state of a non-flowing hard gel. Then, a few gel samples were placed on the rheometer to perform the oscillation test. A polymer gel sample was put between two stationary 40-mm-diameter parallel Peltier plate geometries, with the upper cone spinning at a 4° angle. There was a 1.0 mm space between them. All measurements were taken at ambient temperature. When the measurements began, the rheometer automatically generated the elasticity data.

According to Al-Muntasheri et al., gel strength is associated with gel crosslinking density [23]. In general, oscillation tests determine the viscoelasticity or numerical strength value of a material as a function of time, temperature, or frequency. The variation in the storage modulus (G′) and the energy dissipation or loss modulus (G″) primarily show the viscoelasticity of the polymeric gel. As a general rule, G′ denotes the elasticity of the gel system, whereas G″ denotes viscous behavior. G′ is larger than G″ in a firmly formed polymer gel, indicating that the elastic effect is dominant. It denotes a polymeric gel’s solid-like properties. On the other hand, if G″ is greater than G′, the viscous effect becomes more dominant; hence, it indicates a polymer gel’s liquid-like properties.

There are two kinds of oscillation testing: strain sweep tests and frequency sweep tests. These tests can evaluate whether a polymer gel is viscous or elastic across a certain range of strain or angular frequency. The strain sweep test is theoretically used to assess the region of linear viscoelastic response (LVR) of tested materials. The measured viscoelasticity values inside the LVR do not exhibit significant changes, indicating that strain is negligible. When the applied stress becomes too great, the produced strain begins to disrupt the elastic structure of the tested materials. In this situation, the experimental work must be performed with the strain below its critical point to ensure that the tested samples are inside the LVR range.

The strain sweep measurements in this investigation were performed by evaluating the dynamic viscoelastic moduli at a constant frequency of 1.0 rad s^−1^ and gradually increasing strain in the range of 0.1 to 100%. The frequency sweep, on the other hand, examines the response of the material’s viscoelastic characteristics to the applied frequency rate to imitate the polymer solutions at rest condition. Measurements are taken in frequency sweep mode over a range of oscillation frequencies while maintaining the oscillation strain and temperature. G′ and G″ were measured as a function of frequency in the range of 0.1 to 100 rad s^−1^ at a constant strain of 1.0% and an ambient temperature of 25 °C in this investigation.

### 2.4. Coreflooding Test

The coreflooding test without crossflow effect was conducted in this research utilizing native Berea sandstone core samples. Four sets of polymer gelant were prepared and subsequently injected into the core samples. Table 1 summarizes the injection scenarios that have been conducted in this study. These gelant samples are adequate to simulate the influence of salinity, retarder, and solid nanoparticles on the gel in porous media.

#### 2.4.1. Core Preparation

The Berea sandstone core was used as the porous media for coreflooding experiments. The core’s approximate dimensions were 3.8 cm (1.5 inch) in diameter and 7.6 cm in length (3 inches). This investigation used four Berea core plugs with permeability ranging from 300 to 350 mD. All core samples were cut from the same block of outcrop (open cut mine) rocks that contained no oil. The core samples were extracted using a drilling core sampling device. The extracted core samples were dried for 24 h in a hot oven at 100 °C until their weight was constant. Then, the dry weight, length, and diameter of the core plugs were measured. In addition, a porosimeter and permeameter (Poroperm) were then employed to measure the porosity and gas permeability of the core samples, respectively. Table 2 shows the measured dimensions and average petrophysical attributes of the core samples. The last procedure in core sample preparation involved the core samples being saturated with brine (30,000 ppm NaCl) using a manual saturator under 1000 psi of pressure. This saturation method followed the American Petroleum Institute standard (API RP 40).

#### 2.4.2. Coreflooding Setup

**Figure 3:**
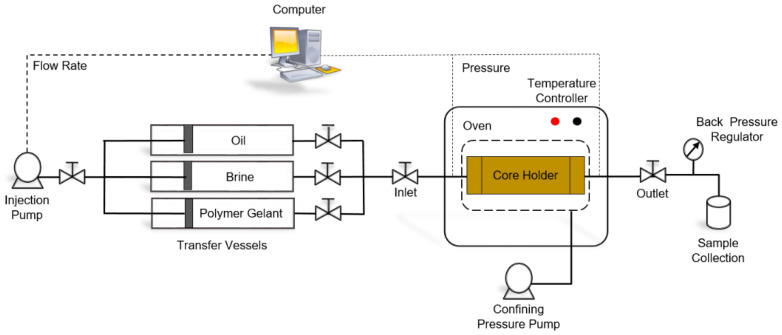
Schematic diagram of the coreflooding set-up.

**Figure 4:**
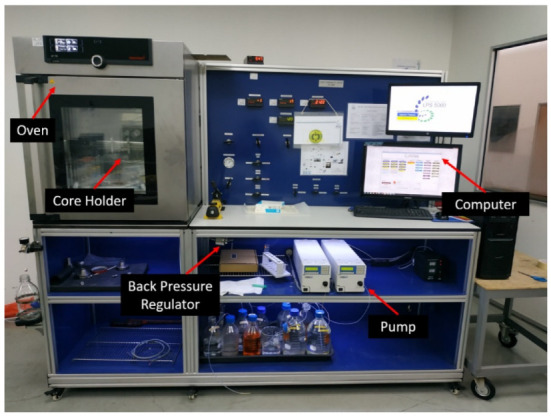
Photo of the coreflooding set-up.

Figure 3 and Figure 4 show a schematic diagram and a photo of the coreflooding setup used to evaluate the blocking capabilities of PAM/PEI polymer gels in porous media. The major components of the experimental apparatus included a core holder designed to replicate reservoir formation, as well as a constant-rate HPLC pump for the injection of brine and polymer gelant. The displacement tests were carried out in a triaxial core holder consisting of a stainless steel (SS316L) cylinder. The core holder was put into an oven for high-temperature exposure. Furthermore, pressure transducers were linked to the inlet and outlet ends of the core holder to monitor and record the differential pressure or pressure drop along the core during the experiment. The output data of the transducers were then sent to the computer as pressure readings. The computer also communicated with the pump in order to record the injection pressure, cumulative injected volume, and flow rate as a function of time. All data were shown in real time and saved to a datasheet.

#### 2.4.3. Coreflooding Procedure

The core was inserted into a core holder that was already in a convection heating oven with ± 1 °C precision. The coreflooding tests were carried out at a constant temperature of 120 °C and the confining pressure was set at 2000 psi. The injection flow rate and pressure of the fluids were kept constant at 0.5 cm^3^/min and 1500 psi, respectively. The injection flow rate was chosen because it was within the range of shear rates commonly used in water flooding operations at the oilfield. In addition, 1500 psi back pressure was applied. Aside from balancing injection pressure, another function of back pressure is to prevent liquid evaporation. It is linked to a data gathering system, which can record data points for all parameters every 30 s. The displacement tests were carried out in sequential flooding procedures after the cores had been mounted in the core holder. To begin, brine was injected into the core at various flow rates to determine the absolute permeability. Darcy’s law of linear flow was used to calculate it. This initial brine injection will also achieve initial water saturation (S_wi_). Following this, as a conformance control mode, the core was continuously flooded with the PAM/PEI polymer gelants. The core was shut-in for three days after the injection of polymer gelants to allow the gelling process to complete. Following gelation, post-treatment water flooding was performed, and the differential pressure versus time was measured.

## 3. Results and Discussion

### 3.1. Gelant Viscosity

**Figure 5:**
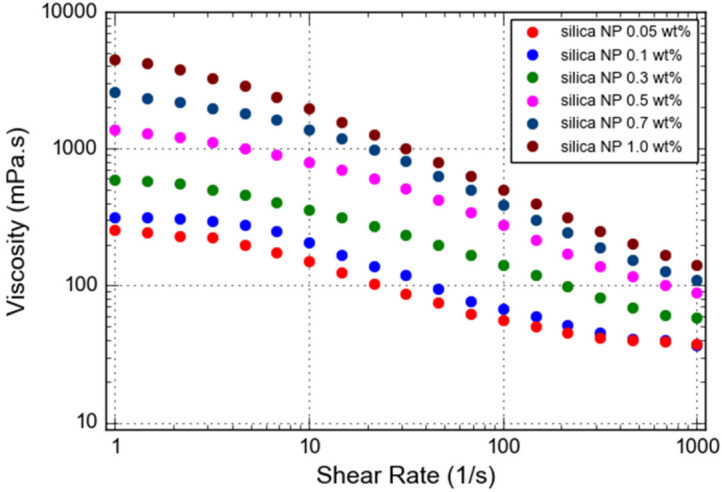
Viscosity as a function of shear rate for PAM/PEI polymer gelant reinforced with different silica NP amounts.

PAM/PEI polymer gel strengthened with silica nanoparticles (silica NP) that acted as a solid filler was studied. This was an improvement of the gel strength, which has been identified as a disadvantage of PAM/PEI polymer gel containing NH_4_Cl in high salinity. The viscosity of the polymer gelant reinforced with silica NP was larger than the viscosity of the polymer gelant without silica NP at the same salinity, retarder content, and shear rate. The addition of silica NP in the PAM/PEI gelling solution led to an increase in viscosity. The results can be observed in Figure 5. The addition of silica NP by more than 0.5 wt% is equivalent to the viscosity of pure PAM/PEI polymer gelant. This compensates for the low viscosity due to the high salinity and retarder content in PAM/PEI polymer gelant. Strong interaction between polymer and nanoparticles is believed to be responsible for this pattern. According to Kamibayashi et al., the ion–dipole interaction is developed between cations and oxygen atoms in the tetrahedral structure of silica [24]. In the presence of silica NP, the inclusion of cations on the carboxylate groups of polymer molecules is decreased to some extent, and the viscosity of the solution increases. In fact, the irreversible adsorption of nanoparticles on a polymer results in a stable and difficult-to-break macro-molecular structure.

Furthermore, the viscosity of the solution was clearly maintained at low shear rates. This is due to the fact that the bond between the polymer and nanoparticles is not simply broken in the normal state, but gradually weakens when the shear rate increases, causing the gelant to demonstrate shear thinning behavior. However, at a high shear rate, the solution seems to lose its non-Newtonian quality and shows less shear thinning behavior. Based on this finding, adding a small amount of nanoparticles to a polymer solution can enhance its pseudo-plasticity behavior at a given shear rate. However, the optimum amount of silica NP should be considered to avoid drastic increments in viscosity that may affect the propagation of the polymer gelant into the target zone.

### 3.2. Gelation Time

**Figure 6:**
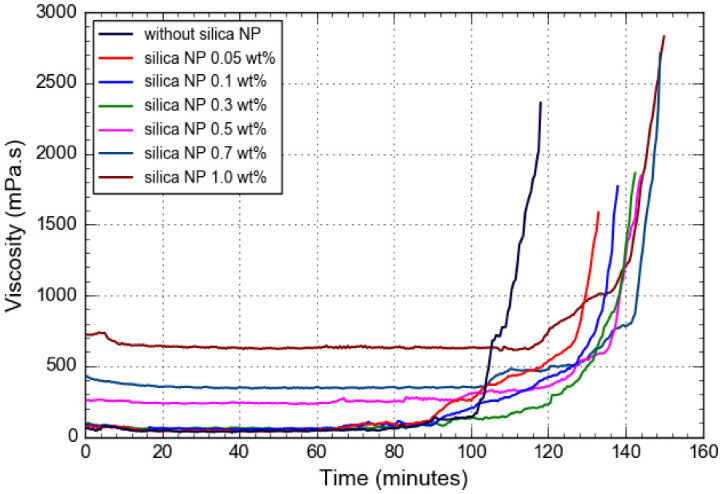
Viscosity changes as a function of time for PAM/PEI polymer gel with different concentrations of silica NP.

**Figure 7:**
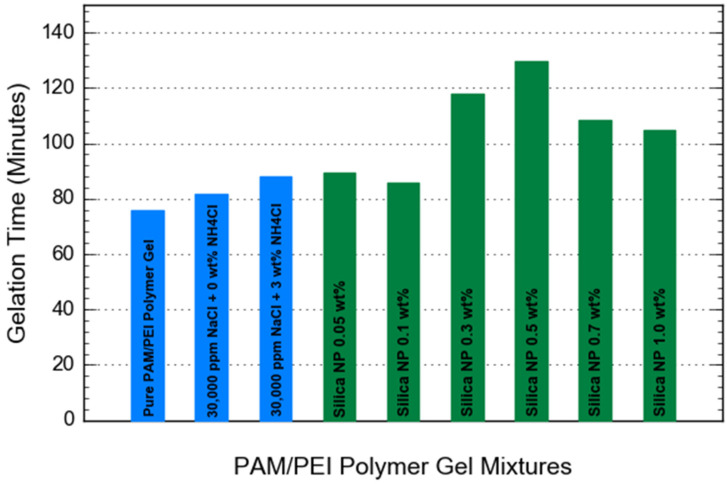
Summary of gelation time for PAM/PEI polymer gel with different concentrations of silica NP.

The effect of silica NP on the gelation time was evaluated by examining the apparent viscosity changes of gelling solutions at 95 °C prepared with various concentrations of silica NP. Results are shown in Figure 6. As information from the previous section shows, the gelation of the pure PAM/PEI gel started at 76 min, while the gelation kinetics of the PAM/PEI gel containing 0.5 wt% NH_4_Cl at salinity 30,000 ppm NaCl began after 80 min. From the summarized result in Figure 7, the PAM/PEI gels containing various amounts of silica NP displayed different responses regarding the gelation time. It can be seen that the gelation time of PAM/PEI gel containing 0.05 and 0.1 wt% silica NP began after 80 min. Even though there were increments in gelation time, the increment seemed less significant compared to the pure PAM/PEI gel and PAM/PEI gel with retarder in high salinity. This observation suggests that the addition of silica NP at a lower concentration does not prolong the gelation time as effectively as the retarder.

According to some literature, a lower concentration of solid particles could open the crosslinking sites more rapidly, thus allowing the polymer chain to crosslink faster with the crosslinker [17,25]. Furthermore, the gelation times of PAM/PEI gels filled with 0.3 and 0.5 wt% silica NP are 118 and 129.5 min, respectively, which are longer than the gelation time of PAM/PEI gel filled with a lower concentration of silica NP. This observation is expected as the silica NP increases; the gelation should increase further. The possible explanation for this behavior could be that the incorporation of 0.3 and 0.5 wt% silica NP made the crosslinking sites unavailable for PEI and PAM to react on time, and this resulted in a delay in the gelation time. This phenomenon is supported by the report by Adewunmi et al., when their PAM/PEI polymer gel reinforced with coal fly ash (CFA) at a concentration of more than 1.0 wt% showed a longer gelation time [25].

However, when the gelling solution is prepared with a higher concentration of silica NP, the gelation time is significantly reduced. The gelation time decreases from approximately 120 min at silica NP content 0.5 wt% to around 105 min when the polymer gels are added with 0.7 and 1.0 wt% of silica NP, respectively. The findings might be attributed to the effect of silica nanoparticles on the gel structure. When silica NP are added to the gelling solution, a significant amount of nanoparticles is doped into the polymer coils. As the gelation process continues, the nanoparticles aggregate together and create microscopic structures. This raises the apparent viscosity of the gelling solution [26]. This leads to a shorter gelation time for gelling solutions containing silica NP.

### 3.3. Gel Strength

**Figure 8:**
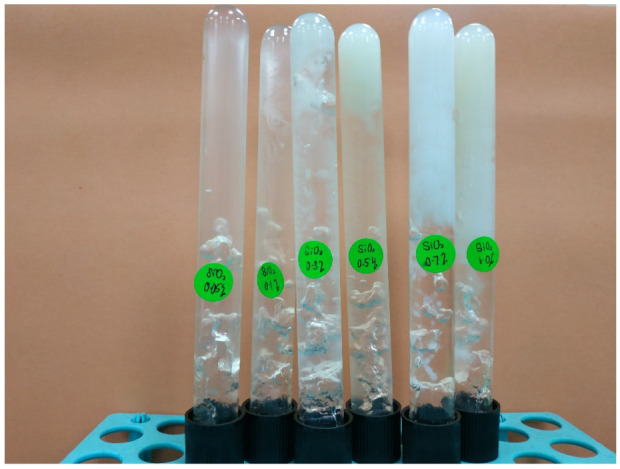
Photo of the formed PAM/PEI polymer gel with different concentrations of silica NP at retarder 5 wt% and salinity 30,000 ppm.

After determining the gelation time, the rheological property of a polymeric composite gel is particularly important for fundamental research, because it allows us to understand the strength, microstructure, and dispersion of solid particles in a gel matrix. When the sample formed mature gels as shown in Figure 8, dynamic rheological experiments were utilized to determine the gel strength of polymer gels reinforced with silica NP.

*a.*  *Frequency-Sweep*

**Figure 9:**
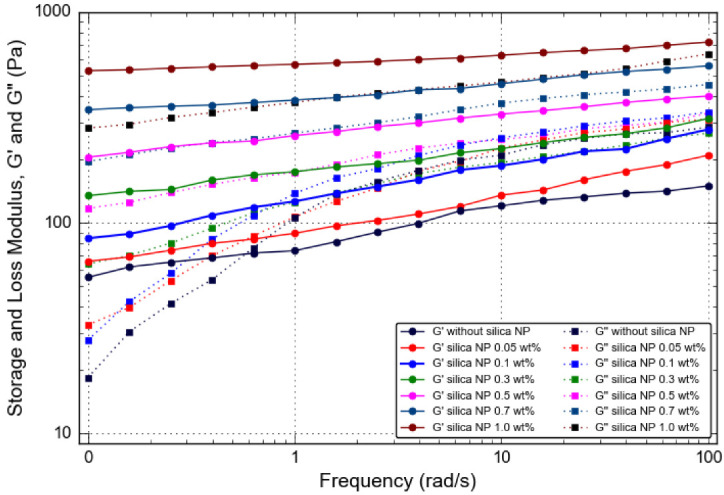
Storage and loss modulus, G′ and G″, as a function of frequency for PAM/PEI polymer gel with different concentrations of silica NP.

Figure 9 illustrates the viscoelastic curves (storage modulus, G′, and loss modulus, G″) as a function of angular frequency ranging from 0.1 to 100 rad/s for the gel samples prepared with silica NP up to 1.0 wt% at retarder 5 wt% and salinity 30,000 ppm, obtained using oscillatory rheology measurements. The results exhibited an apparently very high storage modulus, G′, compared to loss modulus, G″, for gel incorporated with silica NP throughout the whole frequency range, indicating solid-like behavior. Due to this trend, it implies that the elastic character is dominant in the rheological characteristics of reinforced polymer gels. This is in contrast with the case of PAM/PEI gels containing retarder in high salinity, which displayed fluid-like behavior (G″ > G′), especially at high frequency, more than 10 rad/s. In fact, the G′ of PAM/PEI gels containing silica NP is always higher than the G′ of PAM/PEI gel without silica NP. The result indicates that silica NP significantly improves the elasticity of the gel that has been weakened by salinity and retarder. In addition, the G′ of PAM/PEI polymer gel containing silica NP increases at the frequency range 0.1–10 rad/s. However, at higher frequencies of more than 10 rad/s, G′ does not show significant changes, especially at higher silica NP content. It was also observed that G″ demonstrated a very weak dependency on frequency over the range 0.1–10 rad/s and reached a plateau modulus when the frequency was more than 10 rad/s. This observation is more significant for the polymer gel containing a higher amount of silica NP as well. This finding supports the formation of a rubbery gel structure. This result is almost similar to the previous experimental works embedding solid particles in a polymer gel [17,25,27]. For example, Adewunmi et al. reported that their CFA-reinforced polymer gel illustrated rubber-like behavior at a high frequency range, and liquid-like behavior at a low frequency range [28].

This observation is in contrast to the gel containing retarder in high salinity, which demonstrated a strong dependency of G′ on frequency, thus showing weak gel strength. It is also obvious that both G′ and G′’ increase with the increase in silica NP added to the gelling solutions. In fact, it is worth noticing that the ratio of G′ and G″ was more apparent as silica NP increased. Even though the small amount of silica NP did not increase the G′ values, the improvement was more significant at higher amounts of silica NP. This result can be seen in Figure 9: when the silica NP is more than 0.5 wt%, G′ increases at a higher magnitude.

A higher silica NP concentration contributes to more silica NP being filled and arranged in the three-dimensional network structure of gels. This is in accordance with the macroscopic gel strength for gels prepared with solid material. The addition of silica NP changes the rheological properties of the gel, making it more solid-like. The improved viscoelastic characteristics of these gelants demonstrate the excellent interaction and strong bonding between the silica NP and PAM/PEI molecule chains. It also demonstrates that the PAM/PEI molecular chain is not disrupted by the addition of silica NP. The reinforcement by silica NP is effective even for polymer gels with high NH_4_Cl content and in high salinity. Thus, a strong and rigid polymer gel for water shut-off can be produced.

*b.*  *Strain-Sweep*

**Figure 10:**
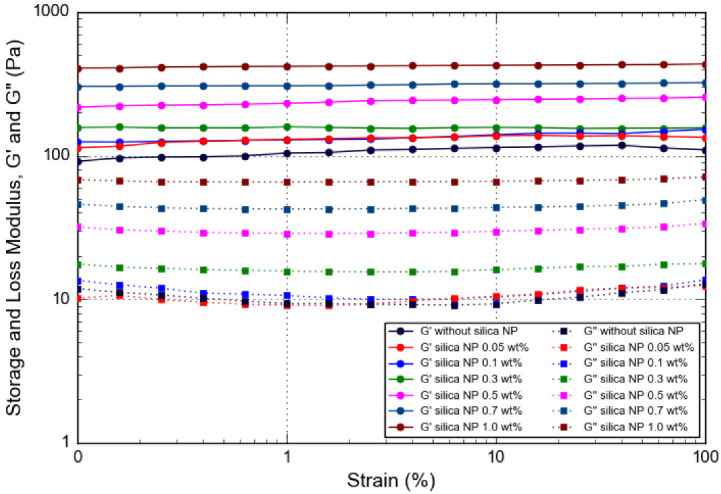
Storage and loss modulus, G′ and G″, as a function of strain for PAM/PEI polymer gel with different concentrations of silica NP.

The strain-sweep measurements were carried out to examine the effect of large deformation on crosslinked polymer gel with various silica NP concentrations. Figure 10 shows the dynamic rheological data (G′ and G″) for PAM/PEI polymer gels without silica nanoparticles and PAM/PEI composite gels reinforced with different amounts of silica NP at retarder 5 wt% and salinity 30,000 ppm against strain. As with the results of frequency sweep, for the PAM/PEI polymer gel with silica NP, G′ is higher over G″ across the whole range of strain amplitude, indicating solid-like behavior. In addition, the G′ values of PAM/PEI-silica NP gels were completely independent of the applied strain up to 100%. The strain dependence of G′ signifies a region of viscoelasticity with a plateau in G′ nearly constant throughout the strain amplitude. This plateau region can be concluded as the linear viscoelastic region (LVR) for PAM/PEI polymer gel reinforced with silica NP. Moreover, the G′ and G″ values of PAM/PEI-silica NP composite gels increase as the silica NP concentration increases. Hence, the result confirms that PAM/PEI polymer gels embedded with higher silica NP content demonstrate higher G′ and G″ within LVR compared to the PAM/PEI gel without silica NP. As silica NP increases, the gels have a greater ability to resist higher pressure without breakage; thus, they are appropriate for water shut-off applications. This study also reveals that the silica NP concentration should be kept higher than 0.5 wt% to obtain sufficient gel strength to compensate for the weak gel strength when the gel contains retarder and in high salinity.

Structural variations within the gel sample contribute to the strengthening mechanism of silica NP on the gel strength. Basically, a large amount of nanoparticles will be doped into the polymer coils. This occurs when the nanoparticles aggregate together and arrange themselves with particular rules during the crosslinking phase. Aggregations and arrangements occur primarily in polymer chain bunches and gel structure meshes. It efficiently improves the structural strength of the gel. The addition of silica NP to the gelling solution thereby reinforces the gel strength. According to Liu et al., the thermal stability of the gel also can be strengthened with the addition of silica NP into the gelling solution [17]. The surface of silica NP contains a large number of hydroxyl groups (−OH). The hydroxyl groups create bonds with water molecules through hydrogen bonds, forming hydronium ions. These hydronium ions are held together by electrostatic attractions. Additionally, the silica NP contains negative charges in the gelling solution. The hydrogen bonding and electrostatic attraction can both lead to a greater bound water ratio. The greater bound water ratio indicates that the gel is more hydrophilic. This characteristic improves the water holding capacity and thermal stability of the reinforced gel compared to the gel that is prepared without silica NP. Thus, silica NP is effective to strengthen not only the rheological strength of the gel, but also the thermal stability.

### 3.4. Coreflooding Test

**Figure 11:**
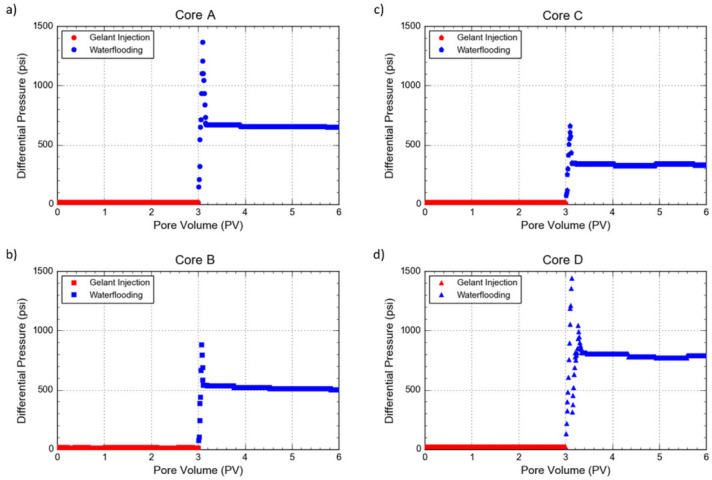
Differential pressure as a function of pore volume in the coreflooding experiments without crossflow effect in Core A (**a**), Core B (**b**), Core C (**c**) and Core D (**d**).

As 0.5 wt% of silica NP is the optimum concentration to achieve sufficient gel strength to compensate for the weakened gel, this concentration was applied for the coreflooding test. This silica NP-reinforced polymer gelant was injected into core #D. The parameter that was evaluated in the coreflooding test was the differential pressure. Differential pressure can be used as an indication of the strength of a gel in blocking the water in porous media. As shown in Figure 11, in general, the differential pressure continues to develop for a short period of time until it achieves its maximum, then decreases towards a plateau. The obvious trends can be observed in the initial stage of post-gelation waterflooding, especially around the first 10 min, where the differential pressure increases in a steady fashion over time. According to the theory and discussions by Zitha et al. and Al-Muntasheri, three mechanisms have been established to explain these trends [29,30]. The initial pressure build-up is the result of the elastic compression of the gel into the porous media. Then, the microscopic flow through the gel becomes the limiting factor for the continuous pressure build-up. It can be understood that when the gel micro-permeability is sizeable, the micro-flow begins to plateau, without gel break-up. Meanwhile, if the micro-permeability is minimal, the critical rupture pressure of the gel is caused, which will form enough channels, and brine is able to pass through the existing channel. This could trigger the macroscopic brine–gel displacement, thus eventually bringing about the final theory, which is the macroscopic displacement of gel by brine. Once the brine breaks through the gel, the macroscopic displacement exerts a less significant impact on the differential pressure. As a result, the final differential pressure tends to plateau.

It can be seen in Figure 11a that, after the gelation of pure PAM/PEI gel in the core, the injection pressure rapidly increased to approximately 1300 psi and then dropped steadily before stabilizing at 650 psi during waterflooding. This obtained differential pressure value is almost in a similar range as the values reported from the study by Al-Muntasheri [30]. They reported that when Bentheimer core samples were treated with PAM/PEI polymer gel, the pressure increased to 1100 psi without initiating flow across the core. The sudden increasing trend of differential pressure could be attributed to the presence of gel blockage within the pore space inside the porous medium, subsequently compressed by brine. As a result, it causes an increasing differential pressure across the core by injecting more brine. Due to the gel deformation, few micro-channels are created in the gel pack for brine to pass through, thus stabilizing the differential pressure, even though a greater pore volume of brine is injected. Therefore, this outcome confirms the theory of Zitha et al. that maximum critical pressure takes place before the differential pressure is stabilized [29].

The highest differential pressure can be observed in the core treated with silica NP-reinforced PAM/PEI polymer gel. The results in Figure 11d show that the differential pressure during waterflooding after gel treatment rose up to 1500 psi. Due to the fact that the pressure was close to the equipment limiting pressure, the pressure was released to around 300 psi of differential pressure. Then, the waterflooding resumed, where the differential pressure increased again to around 1100 psi, before dropping and stabilizing at 800 psi. This shows that the composite polymer gel possesses good gel strength in reducing the permeability and plugging the core, attributed to the solid-like behavior of the gel strengthened with silica NP. Because the gel is filled with silica NP, the gel has a greater ability to resist higher pressure without breakage. On the other hand, the lowest differential pressure is denoted when the treatment by polymer gel contains retarder in high salinity. As depicted in Figure 11c, during waterflooding, the differential pressure across the core increased sharply to around 700 psi, then dropped and reached the plateau value of 350 psi. This trend is lower than the value from the coreflooding work by Al-Muntasheri et al. when the Indiana limestone core was treated with PatBA/PEI polymer gel containing NH_4_Cl [31]. In their study, the differential pressure across the core was maintained at 1000 psi due to the blocking effect of the PAtBA/PEI polymer gel containing retarder prepared with low-salinity water. This is expected since the polymer gel containing retarder prepared in high salinity has lower gel strength. This indicates the deformability of the gel containing retarder compared to the other gels. The low gel strength will be compressed and unable to resist the breakage, thereby promoting lower effectiveness in blocking the flow of water.

This experimental work also proves the thermal stability of the gel even after long hours of high-temperature exposure. All mixtures of PAM/PEI gelant were injected into the cores under high-temperature and high-pressure conditions. Then, the cores were shut-in for 3 days at 120 °C in order to allow the gel to mature. After the shut-in, brine was flooded through the treated core to simulate a production well cycle. As a result, no water discharge was noticed through the core even after 3 days of thermal treatment. This indicates that all PAM/PEI polymer gels were stable and effective in reducing the permeability of Berea sandstone cores, even at 120 °C and under differential pressure of around 1000 psi. The unstable gel system can be easily degraded in the formation and will not effectively block the high-permeability zones. At high temperatures, the heat accelerates the molecular interaction, which breaks the chemical bonds of the gel network. However, this disadvantage is not observed in PAM/PEI polymer gel. Within this gel, the energy of covalent bonds in the gel system is strong enough to overcome the force of intra- and intermolecular reactions and heat exposure. In addition, the network structure of the gel has greater water conservation capacity, which results in minimum syneresis and promotes higher thermal stability. Thereby, organically crosslinked PAM with a PEI gel system, even with the presence of NH_4_Cl and in high salinity, maintains its long-term stability at a temperature of 120 °C.

## 4. Conclusions

From this study, it can be concluded that in order to strengthen the weakened PAM/PEI polymer gel containing retarder in high salinity, the addition of solid particles, namely silica nanoparticles, is considered an effective remedy. At an optimum concentration of 0.5 wt%, silica NP could yield a significant impact in delaying the gelation time. It is apparent that gel strength increases with the increase in silica NP added to gelling solutions, particularly at high amounts of silica NP, thus making it more solid-like. This result indicates the significant improvement in the elasticity of the gel that has been weakened by salinity and retarder. In fact, as silica NP increases, the gel is able to resist higher pressure without breakage, and thus is appropriate for water shut-off applications. The strengthening mechanism is by the nanoparticles increasing and forming arrangements largely in the polymer chain. Regarding the performance in porous media, the PAM/PEI polymer gel strengthened with silica NP has good gel strength in reducing the permeability, and the gel has the ability to withstand higher pressure without collapse.

## Figures and Tables

**Table 1:** The core and injected polymer gelant.

Core Sample	Injected Gelant
A	Pure PAM/PEI polymer gelant
B	PAM/PEI polymer gelant containing 5 wt% retarder
C	PAM/PEI polymer gelant containing 5 wt% retarder at 30,000 ppm salinity
D	Silica NP reinforced PAM/PEI polymer gelant containing 5 wt% retarder at 30,000 ppm salinity

**Table 2:** Petrophysical properties of cores used for coreflooding test.

Core	Length (cm)	Diameter (cm)	Weight (gram)	Porosity	K_air_ (mD)
A	7.324	3.828	176.319	20.336	332.312
B	7.49	3.83	179.651	20.367	364.815
C	7.6	3.84	182.434	20.26	367.12
D	7.324	3.83	176.079	20.48	364.369
